# 4-Methyl-*N*′-(2-oxoindolin-3-yl­idene)benzene-1-sulfono­hydrazide

**DOI:** 10.1107/S1600536811046605

**Published:** 2011-11-12

**Authors:** Alexandra de Souza Fonseca, Tomás Garcia Storino, Vanessa Santana Carratu, Aline Locatelli, Adriano Bof de Oliveira

**Affiliations:** a Escola de Química e Alimentos, Universidade Federal do Rio Grande, Av. Itália km 08, Campus Carreiros, 96201-900, Rio Grande-RS, Brazil; b Departamento de Química, Universidade Federal de Santa Maria, Av. Roraima, Campus, 97105-900, Santa Maria-RS, Brazil; c Departamento de Química, Universidade Federal de Sergipe, Av. Marechal Rondon s/n, Campus, 49100-000, São Cristóvão-SE, Brazil

## Abstract

In the title compound, C_15_H_13_N_3_O_3_S, the C—S—N(H)—N linkage is non-planar, the torsion angle being −65.12 (13)° and the S atom showing a tetra­hedral environment. The compound has two almost planar fragments linked to the S atom: the isatin-derivative fragment [(C_8_H_5_NO)N—N(H)–] and the tolyl fragment [C_7_H_7_–] have maximum deviations from the mean plane through the non-H atoms of 0.0813 (13) and 0.0094 (16) Å, respectively, and make an inter­planar angle of 80.48 (3)°. In the crystal, mol­ecules are connected into inversion dimers *via* pairs of N—H⋯O hydrogen bonds. Additionally, the mol­ecular structure is stabilized by an intra­molecular N—H⋯O hydrogen bond.

## Related literature

For the synthesis of isatin-3-tosyl­hydrazone, see: Cava *et al.* (1958 ▶). For the anti­fungal and anti­bacterial properties of isatin derivatives, including the title compound, see: Chohan *et al.* (2004 ▶).
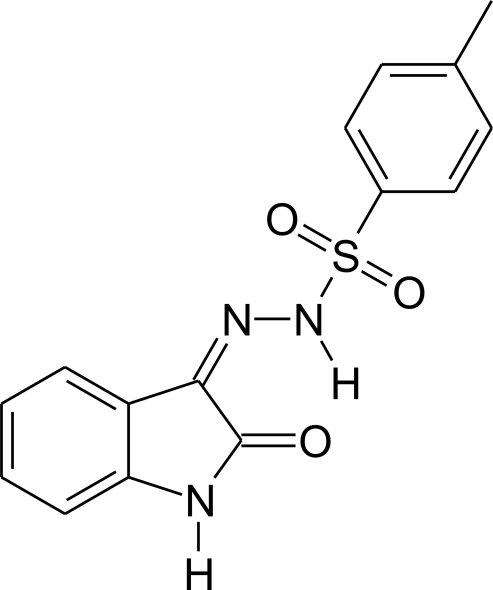


## Experimental

### 

#### Crystal data

C_15_H_13_N_3_O_3_S
*M* *_r_* = 315.35
Monoclinic, 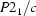

*a* = 14.9050 (3) Å
*b* = 5.7849 (1) Å
*c* = 17.8112 (3) Å
β = 110.427 (1)°
*V* = 1439.18 (5) Å^3^
*Z* = 4
Mo *K*α radiation
μ = 0.24 mm^−1^
*T* = 293 K
0.56 × 0.16 × 0.10 mm

#### Data collection

Bruker X8 APEXII CCD area-detector diffractometer
Absorption correction: multi-scan (*SADABS*; Bruker, 2005 ▶) *T*_min_ = 0.877, *T*_max_ = 0.976
15630 measured reflections
4204 independent reflections
3146 reflections with *I* > 2σ(*I*)
*R*_int_ = 0.026

#### Refinement

*R*[*F* ^2^ > 2σ(*F* ^2^)] = 0.042
*wR*(*F* ^2^) = 0.113
*S* = 1.05
4204 reflections
208 parameters
H atoms treated by a mixture of independent and constrained refinement
Δρ_max_ = 0.28 e Å^−3^
Δρ_min_ = −0.44 e Å^−3^

### 

Data collection: *APEX2* (Bruker, 2005 ▶); cell refinement: *SAINT* (Bruker, 2005 ▶); data reduction: *SAINT*; program(s) used to solve structure: *SHELXS97* (Sheldrick, 2008 ▶); program(s) used to refine structure: *SHELXL97* (Sheldrick, 2008 ▶); molecular graphics: *DIAMOND* (Brandenburg, 2006 ▶); software used to prepare material for publication: *publCIF* (Westrip, 2010 ▶).

## Supplementary Material

Crystal structure: contains datablock(s) I, global. DOI: 10.1107/S1600536811046605/nc2252sup1.cif

Structure factors: contains datablock(s) I. DOI: 10.1107/S1600536811046605/nc2252Isup2.hkl

Supplementary material file. DOI: 10.1107/S1600536811046605/nc2252Isup3.cml

Additional supplementary materials:  crystallographic information; 3D view; checkCIF report

## Figures and Tables

**Table 1:** Hydrogen-bond geometry (Å, °)

*D*—H⋯*A*	*D*—H	H⋯*A*	*D*⋯*A*	*D*—H⋯*A*
N3—H6⋯O1	0.83 (2)	2.08 (2)	2.7539 (17)	138.3 (19)
N1—H1⋯O1^i^	0.86 (2)	2.04 (2)	2.9029 (16)	172.9 (18)

Symmetry code: (i) 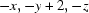.

**Fig. 1.**
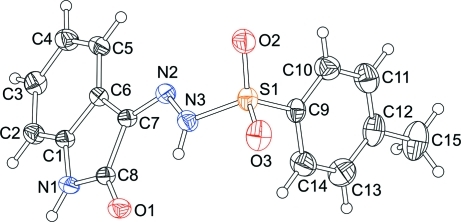
: The molecular structure of the title compound with labeling and displacement ellipsoids drawn at the 40% probability level.

**Fig. 2.**
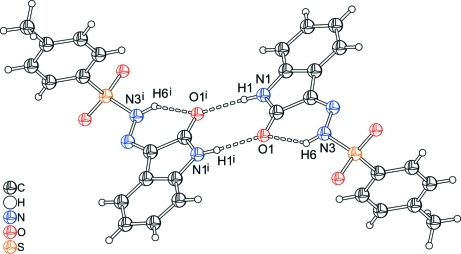
: Crystal structure of the title compound showing the dimers. Intermolecular hydrogen bonding is indicated as dashed lines. Symmetry code: (i)-x, -y + 2, -z.

## supplementary crystallographic information

### Comment

Isatin derivatives have a wide range of biological properties. For example, isatin-based hydrazones show pharmacological activity against bacteria and fungi (Chohan *et al.*, 2004). As part of our study of isatin derivatives, we report herein the crystal structure of isatin-3-tosylhydrazone. In the title compound (Fig. 1) the C—S—N(H)—N linkage is non-planar with the torsion angle being 65.12 (13)° and a tetrahedral environment suggests a *sp*^3^ hybridization for the S atom. The title structure contains additionally two planar fragments. The mean deviations from the least squares planes for the isatin-derivative fragment C1/C2/C3/C4/C5/C6/C7/C8/N1/N2/N3/O1 and for the tolyl fragment C9/C10/C11/C12/C13/C14/C15 amount to 0.0813 (13)° for C2 and 0.0094 (16)° for C11 atoms, respectively, with a dihedral angle of 80.48 (3)°. The crystal packing is stabilized by intermolecular N—H···O (Table 1; N1—H1···O1^i^) and also by intramolecular N—H···O bonds (Table 1; N3—H6···O1) leading the isatin-3-tosylhydrazone dimers (Fig. 2). Symmetry code: (i)*-x, -y + 2, -z*.

### Experimental

Starting materials were commercially available and were used without further purification. The synthesis was adapted from a procedure reported previously (Cava *et al.*, 1958). The glacial acetic acid catalyzed reaction of isatin (5 mmol) and *p*-toluenesulfonylhydrazine (5 mmol) in methanol (60 ml) was refluxed for 5 h. After cooling and filtering, crystals suitable for X-ray diffraction were obtained.

### Refinement

H atoms attached to C atoms were positioned with idealized geometry and were refined isotropic with *U*_eq_(H) set to 1.2 times of the *U*_eq_(C) for the aromatic and 1.5 times of the *U*_eq_(C) for methyl H atoms using a riding model with C—H = 0.93 Å and C—H = 0.96 Å, respectively. H atoms attached to N atoms were located in difference Fourier maps and included in the subsequent refinement using restraints (N1—H1 = 0.86 (2) Å and N3—H6 = 0.83 (2) Å) with *U*_iso_(H) = 1.2 times of the *U*_eq_(N). In the last stage of refinement, they were treated as riding on their parent N atoms.

### Crystal data

**Table:**

C_15_H_13_N_3_O_3_S	*F*(000) = 656
*M**_r_* = 315.35	*D*_x_ = 1.455 Mg m^−^^3^
Monoclinic, *P*2_1_/*c*	Melting point: 476 K
Hall symbol: -P 2ybc	Mo *K*α radiation, λ = 0.71073 Å
*a* = 14.9050 (3) Å	Cell parameters from 4508 reflections
*b* = 5.7849 (1) Å	θ = 2.9–26.0°
*c* = 17.8112 (3) Å	µ = 0.24 mm^−^^1^
β = 110.427 (1)°	*T* = 293 K
*V* = 1439.18 (5) Å^3^	Block, yellow
*Z* = 4	0.56 × 0.16 × 0.10 mm

### Data collection

**Table:**

Bruker X8 APEXII CCD area-detector diffractometer	4204 independent reflections
Radiation source: fine-focus sealed tube, Bruker X8 APEXII	3146 reflections with *I* > 2σ(*I*)
graphite	*R*_int_ = 0.026
φ and ω scans	θ_max_ = 30.1°, θ_min_ = 2.9°
Absorption correction: multi-scan (*SADABS*; Bruker, 2005)	*h* = −20→21
*T*_min_ = 0.877, *T*_max_ = 0.976	*k* = −8→5
15630 measured reflections	*l* = −25→23

### Refinement

**Table:**

Refinement on *F*^2^	Primary atom site location: structure-invariant direct methods
Least-squares matrix: full	Secondary atom site location: difference Fourier map
*R*[*F*^2^ > 2σ(*F*^2^)] = 0.042	Hydrogen site location: inferred from neighbouring sites
*wR*(*F*^2^) = 0.113	H atoms treated by a mixture of independent and constrained refinement
*S* = 1.05	*w* = 1/[σ^2^(*F*_o_^2^) + (0.0476*P*)^2^ + 0.4436*P*] where *P* = (*F*_o_^2^ + 2*F*_c_^2^)/3
4204 reflections	(Δ/σ)_max_ = 0.001
208 parameters	Δρ_max_ = 0.28 e Å^−^^3^
0 restraints	Δρ_min_ = −0.44 e Å^−^^3^

### Special details

**Table:**

Geometry. All e.s.d.'s (except the e.s.d. in the dihedral angle between two l.s. planes) are estimated using the full covariance matrix. The cell e.s.d.'s are taken into account individually in the estimation of e.s.d.'s in distances, angles and torsion angles; correlations between e.s.d.'s in cell parameters are only used when they are defined by crystal symmetry. An approximate (isotropic) treatment of cell e.s.d.'s is used for estimating e.s.d.'s involving l.s. planes.
Refinement. Refinement of *F*^2^ against ALL reflections. The weighted *R*-factor *wR* and goodness of fit *S* are based on *F*^2^, conventional *R*-factors *R* are based on *F*, with *F* set to zero for negative *F*^2^. The threshold expression of *F*^2^ > σ(*F*^2^) is used only for calculating *R*-factors(gt) *etc*. and is not relevant to the choice of reflections for refinement. *R*-factors based on *F*^2^ are statistically about twice as large as those based on *F*, and *R*- factors based on ALL data will be even larger.

### Fractional atomic coordinates and isotropic or equivalent isotropic displacement parameters (Å^2^)

**Table:**

	*x*	*y*	*z*	*U*_iso_*/*U*_eq_	
C3	0.11277 (11)	1.0172 (3)	0.32467 (9)	0.0394 (4)	
H3	0.1057	1.1188	0.3627	0.047*	
C11	0.53807 (13)	0.3670 (4)	0.17689 (12)	0.0566 (5)	
H8	0.5908	0.3119	0.2187	0.068*	
C12	0.54616 (13)	0.5683 (4)	0.13898 (12)	0.0527 (5)	
C10	0.45279 (12)	0.2441 (3)	0.15401 (11)	0.0490 (4)	
H7	0.4482	0.1086	0.1805	0.059*	
C13	0.46719 (15)	0.6454 (4)	0.07640 (14)	0.0606 (5)	
H9	0.4717	0.7815	0.0501	0.073*	
C14	0.38184 (13)	0.5253 (3)	0.05197 (12)	0.0540 (5)	
H10	0.3296	0.5786	0.0094	0.065*	
C15	0.63816 (15)	0.7038 (5)	0.16498 (16)	0.0760 (7)	
H11	0.6298	0.8449	0.1901	0.114*	
H12	0.6553	0.7391	0.1191	0.114*	
H13	0.6881	0.6138	0.2023	0.114*	
H6	0.1551 (15)	0.431 (4)	0.0260 (12)	0.059 (6)*	
H1	0.0168 (14)	1.043 (4)	0.0776 (12)	0.055 (6)*	
S1	0.26748 (3)	0.16782 (7)	0.06008 (2)	0.03535 (12)	
N3	0.18364 (9)	0.3472 (2)	0.06478 (8)	0.0352 (3)	
N2	0.19062 (8)	0.4241 (2)	0.13897 (7)	0.0320 (3)	
C8	0.08200 (10)	0.7533 (3)	0.07301 (8)	0.0305 (3)	
O1	0.06596 (8)	0.71841 (19)	0.00121 (6)	0.0382 (3)	
C9	0.37519 (11)	0.3244 (3)	0.09182 (9)	0.0347 (3)	
N1	0.05136 (9)	0.9330 (2)	0.10573 (7)	0.0329 (3)	
C7	0.14177 (10)	0.6049 (2)	0.14176 (8)	0.0292 (3)	
C1	0.08633 (10)	0.9203 (2)	0.19011 (8)	0.0295 (3)	
O3	0.23997 (9)	0.1214 (2)	−0.02344 (7)	0.0511 (3)	
C5	0.17706 (11)	0.6607 (3)	0.29508 (9)	0.0353 (3)	
H5	0.2115	0.5247	0.3121	0.042*	
C2	0.07358 (11)	1.0743 (3)	0.24401 (9)	0.0351 (3)	
H2	0.0400	1.2115	0.2272	0.042*	
C6	0.13941 (10)	0.7169 (2)	0.21428 (8)	0.0298 (3)	
C4	0.16212 (11)	0.8120 (3)	0.34970 (9)	0.0398 (4)	
H4	0.1855	0.7755	0.4040	0.048*	
O2	0.27543 (8)	−0.0145 (2)	0.11553 (8)	0.0481 (3)	

### Atomic displacement parameters (Å^2^)

**Table:**

	*U*^11^	*U*^22^	*U*^33^	*U*^12^	*U*^13^	*U*^23^
C3	0.0415 (8)	0.0398 (9)	0.0366 (8)	0.0005 (7)	0.0132 (7)	−0.0057 (7)
C11	0.0392 (9)	0.0670 (13)	0.0552 (11)	−0.0021 (9)	0.0060 (8)	−0.0073 (10)
C12	0.0403 (9)	0.0587 (12)	0.0630 (12)	−0.0114 (8)	0.0231 (8)	−0.0226 (10)
C10	0.0454 (9)	0.0506 (10)	0.0469 (10)	−0.0021 (8)	0.0109 (8)	0.0040 (8)
C13	0.0556 (12)	0.0479 (11)	0.0792 (15)	−0.0130 (9)	0.0247 (10)	0.0044 (10)
C14	0.0423 (9)	0.0485 (10)	0.0646 (12)	−0.0034 (8)	0.0105 (8)	0.0089 (9)
C15	0.0501 (12)	0.0853 (17)	0.0963 (18)	−0.0272 (12)	0.0300 (12)	−0.0305 (14)
S1	0.0344 (2)	0.0308 (2)	0.0424 (2)	−0.00097 (15)	0.01533 (16)	−0.00615 (15)
N3	0.0354 (7)	0.0364 (7)	0.0338 (7)	0.0062 (5)	0.0122 (5)	0.0009 (6)
N2	0.0333 (6)	0.0304 (6)	0.0335 (6)	0.0027 (5)	0.0133 (5)	0.0016 (5)
C8	0.0282 (6)	0.0315 (7)	0.0314 (7)	0.0007 (6)	0.0101 (5)	0.0068 (6)
O1	0.0424 (6)	0.0417 (6)	0.0294 (5)	0.0047 (5)	0.0111 (4)	0.0051 (5)
C9	0.0322 (7)	0.0333 (8)	0.0411 (8)	−0.0007 (6)	0.0158 (6)	−0.0063 (6)
N1	0.0360 (6)	0.0308 (6)	0.0313 (6)	0.0080 (5)	0.0109 (5)	0.0087 (5)
C7	0.0283 (6)	0.0292 (7)	0.0303 (7)	0.0008 (5)	0.0103 (5)	0.0059 (6)
C1	0.0287 (6)	0.0283 (7)	0.0317 (7)	0.0007 (5)	0.0107 (5)	0.0053 (6)
O3	0.0516 (7)	0.0557 (8)	0.0466 (7)	−0.0074 (6)	0.0177 (6)	−0.0220 (6)
C5	0.0342 (7)	0.0371 (8)	0.0319 (7)	0.0068 (6)	0.0081 (6)	0.0063 (6)
C2	0.0364 (7)	0.0288 (7)	0.0400 (8)	0.0040 (6)	0.0134 (6)	0.0024 (6)
C6	0.0288 (6)	0.0295 (7)	0.0315 (7)	0.0033 (5)	0.0109 (5)	0.0036 (6)
C4	0.0397 (8)	0.0460 (9)	0.0293 (7)	0.0023 (7)	0.0066 (6)	0.0014 (7)
O2	0.0449 (6)	0.0336 (6)	0.0689 (8)	0.0029 (5)	0.0239 (6)	0.0083 (6)

### Geometric parameters (Å, °)

**Table:**

C3—C4	1.385 (2)	S1—N3	1.6488 (13)
C3—C2	1.389 (2)	S1—C9	1.7560 (15)
C3—H3	0.9300	N3—N2	1.3636 (17)
C11—C12	1.372 (3)	N3—H6	0.83 (2)
C11—C10	1.388 (3)	N2—C7	1.2853 (18)
C11—H8	0.9300	C8—O1	1.2329 (17)
C12—C13	1.383 (3)	C8—O1	1.2329 (17)
C12—C15	1.505 (3)	C8—N1	1.3471 (19)
C10—C9	1.373 (2)	C8—C7	1.5060 (19)
C10—H7	0.9300	N1—C1	1.4103 (18)
C13—C14	1.380 (3)	N1—H1	0.86 (2)
C13—H9	0.9300	C7—C6	1.456 (2)
C14—C9	1.383 (2)	C1—C2	1.371 (2)
C14—H10	0.9300	C1—C6	1.3991 (19)
C15—H11	0.9600	C5—C4	1.383 (2)
C15—H12	0.9600	C5—C6	1.3886 (19)
C15—H13	0.9600	C5—H5	0.9300
S1—O2	1.4212 (12)	C2—H2	0.9300
S1—O3	1.4239 (12)	C4—H4	0.9300
			
C4—C3—C2	121.40 (15)	S1—N3—H6	120.1 (14)
C4—C3—H3	119.3	C7—N2—N3	116.77 (12)
C2—C3—H3	119.3	O1—C8—N1	127.08 (13)
C12—C11—C10	121.27 (18)	O1—C8—N1	127.08 (13)
C12—C11—H8	119.4	O1—C8—C7	126.55 (14)
C10—C11—H8	119.4	O1—C8—C7	126.55 (14)
C11—C12—C13	118.32 (17)	N1—C8—C7	106.37 (12)
C11—C12—C15	121.2 (2)	C10—C9—C14	120.46 (16)
C13—C12—C15	120.5 (2)	C10—C9—S1	120.21 (13)
C9—C10—C11	119.39 (18)	C14—C9—S1	119.30 (13)
C9—C10—H7	120.3	C8—N1—C1	111.45 (12)
C11—C10—H7	120.3	C8—N1—H1	122.9 (13)
C14—C13—C12	121.56 (19)	C1—N1—H1	125.6 (13)
C14—C13—H9	119.2	N2—C7—C6	125.84 (13)
C12—C13—H9	119.2	N2—C7—C8	127.94 (13)
C13—C14—C9	118.99 (18)	C6—C7—C8	106.08 (12)
C13—C14—H10	120.5	C2—C1—C6	122.19 (13)
C9—C14—H10	120.5	C2—C1—N1	128.49 (13)
C12—C15—H11	109.5	C6—C1—N1	109.32 (12)
C12—C15—H12	109.5	C4—C5—C6	118.32 (14)
H11—C15—H12	109.5	C4—C5—H5	120.8
C12—C15—H13	109.5	C6—C5—H5	120.8
H11—C15—H13	109.5	C1—C2—C3	117.32 (14)
H12—C15—H13	109.5	C1—C2—H2	121.3
O2—S1—O3	120.64 (8)	C3—C2—H2	121.3
O2—S1—N3	108.22 (7)	C5—C6—C1	119.77 (13)
O3—S1—N3	102.99 (7)	C5—C6—C7	133.54 (13)
O2—S1—C9	108.30 (7)	C1—C6—C7	106.69 (12)
O3—S1—C9	109.34 (8)	C5—C4—C3	120.90 (14)
N3—S1—C9	106.44 (7)	C5—C4—H4	119.5
N2—N3—S1	116.86 (10)	C3—C4—H4	119.5
N2—N3—H6	117.5 (14)		
			
C10—C11—C12—C13	−0.7 (3)	N3—N2—C7—C6	−179.42 (13)
C10—C11—C12—C15	178.77 (19)	N3—N2—C7—C8	−4.3 (2)
C12—C11—C10—C9	0.5 (3)	O1—C8—C7—N2	4.0 (2)
C11—C12—C13—C14	0.1 (3)	O1—C8—C7—N2	4.0 (2)
C15—C12—C13—C14	−179.4 (2)	N1—C8—C7—N2	−174.84 (14)
C12—C13—C14—C9	0.7 (3)	O1—C8—C7—C6	179.91 (14)
O2—S1—N3—N2	51.10 (13)	O1—C8—C7—C6	179.91 (14)
O3—S1—N3—N2	179.90 (11)	N1—C8—C7—C6	1.06 (15)
C9—S1—N3—N2	−65.12 (13)	C8—N1—C1—C2	177.47 (14)
S1—N3—N2—C7	162.65 (11)	C8—N1—C1—C6	−2.60 (16)
N1—C8—O1—O1	0.00 (10)	C6—C1—C2—C3	−2.3 (2)
C7—C8—O1—O1	0.00 (14)	N1—C1—C2—C3	177.61 (14)
C11—C10—C9—C14	0.3 (3)	C4—C3—C2—C1	−0.5 (2)
C11—C10—C9—S1	178.32 (14)	C4—C5—C6—C1	−1.2 (2)
C13—C14—C9—C10	−0.9 (3)	C4—C5—C6—C7	178.95 (15)
C13—C14—C9—S1	−178.95 (16)	C2—C1—C6—C5	3.2 (2)
O2—S1—C9—C10	8.34 (16)	N1—C1—C6—C5	−176.70 (13)
O3—S1—C9—C10	−124.90 (15)	C2—C1—C6—C7	−176.92 (13)
N3—S1—C9—C10	124.50 (14)	N1—C1—C6—C7	3.15 (15)
O2—S1—C9—C14	−173.62 (14)	N2—C7—C6—C5	−6.7 (3)
O3—S1—C9—C14	53.14 (16)	C8—C7—C6—C5	177.26 (16)
N3—S1—C9—C14	−57.46 (15)	N2—C7—C6—C1	173.44 (14)
O1—C8—N1—C1	−177.97 (14)	C8—C7—C6—C1	−2.56 (15)
O1—C8—N1—C1	−177.97 (14)	C6—C5—C4—C3	−1.5 (2)
C7—C8—N1—C1	0.88 (16)	C2—C3—C4—C5	2.4 (3)

### Hydrogen-bond geometry (Å, °)

**Table:**

*D*—H···*A*	*D*—H	H···*A*	*D*···*A*	*D*—H···*A*
N3—H6···O1	0.83 (2)	2.08 (2)	2.7539 (17)	138.3 (19)
N1—H1···O1^i^	0.86 (2)	2.04 (2)	2.9029 (16)	172.9 (18)

Symmetry codes:  (i) −*x*, −*y*+2, −*z*.
